# Emphysematous Epididymo-Orchitis: A Rare Case Report

**DOI:** 10.7759/cureus.38358

**Published:** 2023-04-30

**Authors:** Mohamed Abdelkarim, Abdo Alfares, Hadi Aldarwish, Fatima Albladi, Ahmed Abdelkarim

**Affiliations:** 1 Department of Urology, King Khalid Hospital, Hail, SAU; 2 Department of Surgery, University of Hail College of Medicine, Hail, SAU; 3 Department of Surgery, Sulaiman Al Rajhi University, Al-Qassim, SAU

**Keywords:** gas-producing organisms, infection, gas shadow, epididymo-orchitis, emphysematous

## Abstract

Emphysematous epididymo-orchitis is a rare and potentially fatal infection marked by the presence of gas in the epididymis and testicular tissue. Here, we describe the case of a 49-year-old male with a known past medical history of diabetes and hypertension who presented with right inguinoscrotal swelling and severe tenderness. An urgent scrotal ultrasound was obtained and revealed a fluid-filled avascular mass. Moreover, the non-contrast CT scan showed a mixture of air and fluid density in the right epididymis, perineum, and spermatic cord course. The medical team confirmed the diagnosis of emphysematous epididymo-orchitis. The patient refused the management plan at first, but later came back and accepted the procedure. A right orchidectomy with spermatic cord removal was performed without complications.

## Introduction

Emphysematous epididymo-orchitis is an extremely rare inflammatory process characterized by the presence of gas within the epididymal tissue [1]. There are many conditions reported as possible pathogenesis pathways to develop emphysematous epididymo-orchitis. However, the true pathogenesis is unclear [2]. Diabetes has been a significant risk factor in the case of emphysematous epididymo-orchitis due to urinary tract infections complicated by the presence of gas-producing organisms [3]. Upon searching relevant literature, it was found that there are only a limited number of reported cases of emphysematous epididymo-orchitis [4]. Given the above, we describe the case of a 49-year-old male with a known past medical history of diabetes and hypertension.

## Case presentation

A 49-year-old male presented to the emergency room with painful right testicular swelling for two days associated with pyrexia at 38.5 °C. He had a similar attack four weeks prior. He is circumcised, sexually active (vaginal intercourse), and used a shaver to remove pubic hair, but there are no data on whether or not he used it regularly. He has been a smoker, a diabetic on insulin for three years, and hypertensive for four years. The rest of his vitals were stable on admission. On examination, there was noticeable right-sided swelling that extended from the inguinal area to the upper scrotum, which was tender to palpation. The skin was hot, somewhat darker, and showed crepitus on palpitation. The right testis and epididymis could not be assessed. The rest of the abdomen was non-tender to palpation. There were no urinary symptoms. The patient’s blood showed normal electrolytes, leukocytosis of WBC 23.9 × 109/L (normal 4.5 to 11.0 × 109/L), neutrophils 80% (normal 40% to 60%), hemoglobin 11.5 g/dl (normal 13.5 to 17 g/dl), HbA1c 9.7% (normal 5.7% to 6.4%), and pyuria 30/hpf (normal 0 to 5/hpf).

Relevant investigations

**Figure 1:**
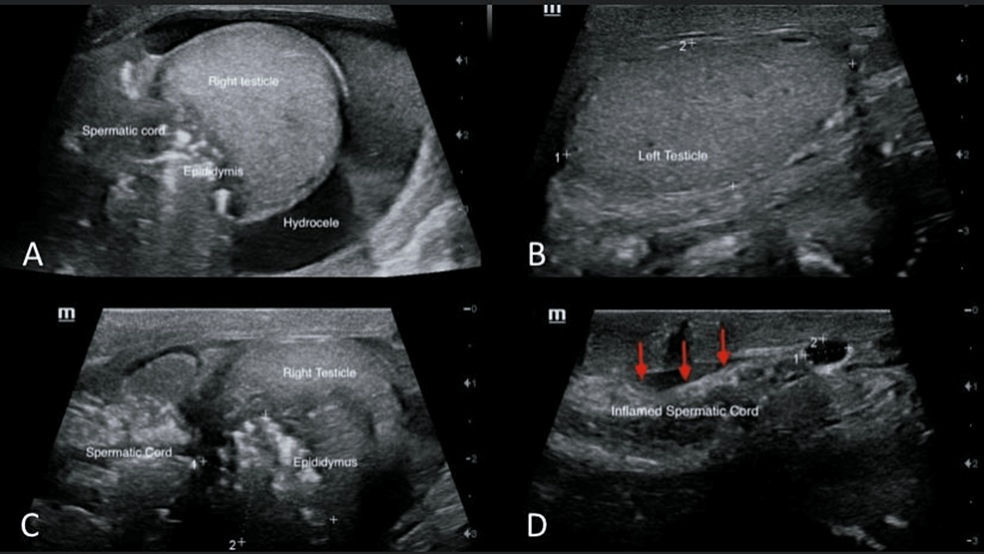
(A) Ultrasound images showing irregular right testis, edematous spermatic cord, enlarged epididymis, and hydrocele; (B) normal left testis; (C) enlarged spermatic cord and epididymis; (D) inflamed spermatic cord.

The scrotal US was performed and showed a grossly enlarged heterogeneous edematous right spermatic cord with intervening hyperechoic fat planes, an irregular contour, and increased vascularity detected within, as well as a markedly enlarged heterogeneous echo pattern of the right epididymal head and body and, to a lesser extent, the right epididymal tail. There are numerous hyperechoic shadows seen along the right spermatic cord and right epidermis (Figure 1).

**Figure 2:**
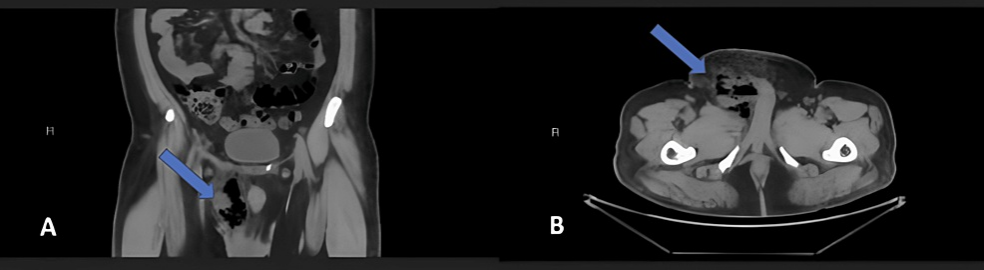
(A) Coronal view of the CT showing mixed air and fluid density seen along the right spermatic cord course and the right epididymis with the air locules seen exceeding the right inguinal canal and the pubic area with subcutaneous air and fat stranding at the right pubic region and (B) axial view showing the same finding.

A non-contrast CT scan of the urinary tract was obtained. The CT revealed there is a mixed air and fluid density seen along the right spermatic cord course and the right epididymis, with the air loculi exceeding the right inguinal canal and the pubic area, with subcutaneous air and fat stranding at the right pubic region. Also, the air density is seen creeping to the right perineal region, with a mild right hydrocele and grossly unremarkable right testis (Figure 2). The described features suggest right scrotal and right perineal gas forming necrotizing fasciitis (Fournier gangrene), which is soon excluded by the non-occurrence of the ordinary blackish discoloration of gangrenous skin.

Surgical events

The patient was counseled regarding the management plan, and the surgical option is recommended. However, the patient declined surgery and was discharged against medical advice (AMA). The next day, the patient presented again and agreed to have surgery.

**Figure 3:**
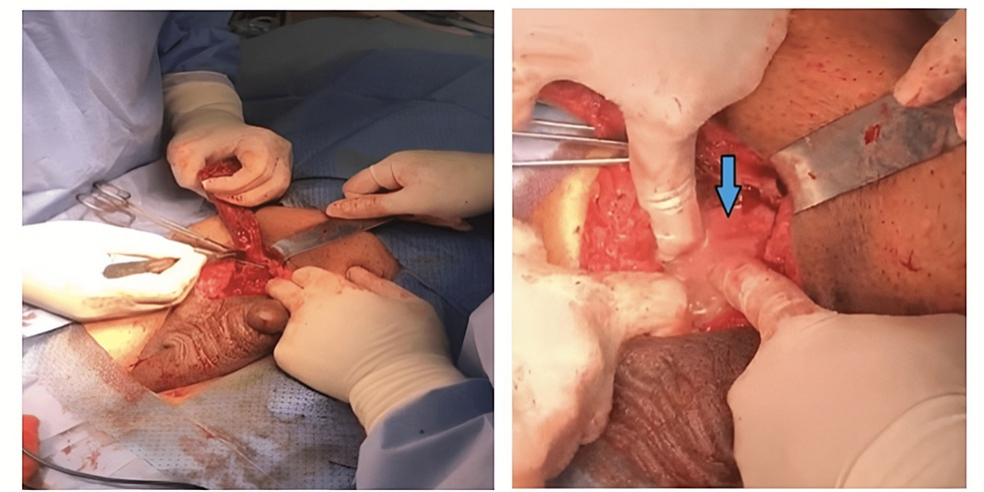
In operation room, there was an extensive abscess with gas forming in the right epididymis, testis, and spermatic cord. A right orchidectomy with a spermatic cord was performed. Blue arrow shows extensive pus coming out of the abscess.

In the theater, the patient was examined under general anesthesia in a supine position, and even a larger swelling was found. Intraoperatively, the right inguinoscrotal incision for exploration of the swollen area revealed both a swollen cord and epididymis (Figure 3). Copious purulent material was coming from the incision; the right testicle, right hemiscrotum, and peritoneal coverings reached all the way up to the internal inguinal ring. A culture swap was done for microbiology. A radical orchiectomy was performed, and a drain (Fr-20) was inserted at the bed of the incision. Hemostasis was ensured, and excision of any remaining necrotic tissues was done. The skin was closed with staples. Following the surgery, the patient was discharged after 10 days due to delayed wound healing and an uncontrolled blood sugar level. He was on piperacillin/tazobactam (tazocin) 4.5 g four times a day (QID) and clindamycin (cleocin) 1 g three times a day (TID) for four days. Four days after being discharged, he returned with a partially healed incisional site and wound discharge. The results for cultures returned showed *Escherichia coli*. The patient was put on meropenem (merrem IV) 1 g TID and amoxicillin/clavulanic acid (augmentin) 1200 mg two times a day (BID) for 14 days after a clinical pharmacist consultation. The patient did not show up for follow-up.

## Discussion

Epididymitis is the most common infection in the scrotum due to the intimate vicinity of the epididymis and the testicular tissue; orchitis would be the expected result in approximately 20-40% of the cases [5,6]. However rare, the presence of gas within the epididymis and the testicular tissue is a pathognomonic feature; only seven cases in the literature were reported [1-7].

The etiology is not exactly understood. However, it is thought to be due to extinction by the refluxed infected urine from other adjacent structures like that which occurs in prostatitis, seminal vesiculitis, or post-operation, direct invasion of the tumor-associated inflammatory process to the urinary tract, and intermittent catheterization that may lead to the extinction of the infection through the vas deferens to the epididymis and testis by the gas forming organisms like *E. coli* or *Klebsiella* causing emphysematous epididymo-orchitis [2,3,5]. In our case, there is still no obvious etiology to this inflammatory process, but the patient is immunocompromised because of his uncontrolled diabetic state and smoking.

Emphysematous epididymo-orchitis manifests as painful swelling of the scrotum, tenderness on palpation associated with fever, and rarely with urethral discharge, dysuria, or pyuria [2,5]. In this case, the patient presented with right inguinal swelling that extended to the right scrotum and severe tenderness without urinary symptoms.

Such infections are usually associated with immunocompromised patients in cases of AIDS or long-term use of steroids, and more often among diabetic patients or due to recurrent UTIs [5,7]. This is identical to this patient because he is immunocompromised because of chronic diseases such as diabetes. In addition to that, he is a heavy smoker.

Imaging plays an important role in the diagnosis, and the US is the most commonly used modality in scrotal and intra-scrotal lesions. This may show gas locules in emphysematous epididymo-orchitis appearing within the parenchyma as hyperechoic areas, which are considered pathognomonic, and alteration and/or enlargement of the echotexture [4-6]. CT is preferred to confirm the diagnosis due to its high sensitivity and specificity in detecting the presence of gases. In young patients, MRI should be considered despite its high cost and time consumption to avoid radiation exposure to the testis [4,5].

The differential diagnosis of acute scrotal presentation, including Fournier’s gangrene, epididymo-orchitis, testicular torsion, testicular shrapnel, or testicular tumor, must be distinguished because their treatment significantly differs [1,4,6]. All of these should be considered for any patient who presents with inguinoscrotal swelling and tenderness.

This infection is a life-threatening condition, and it is a surgical emergency because it may complicate to cellulitis, which in turn predisposes to septic shock and/or necrotizing fasciitis, which might lead to death [4,7]. Fortunately, the team intervened before the occurrence of any complications.

The mainstay treatment for emphysematous epididymo-orchitis is antibiotic therapy, drainage of pus, and orchidectomy with surgical debridement [1,4]. However, our patient received piperacillin/tazobactam (tazocin) and clindamycin (cleocin) as empirical antibiotics for four days, then changed to meropenem (merrem IV) and amoxicillin/clavulanic acid (augmentin) according to culture sensitivity test results.

## Conclusions

This is a case of emphysematous epididymo-orchitis in a smoker, diabetic, and hypertensive patient who presented with right inguinoscrotal swelling with severe tenderness on examination. Urgent ultrasound and computed tomography of the kidneys, ureters, and bladder (KUB CT) scans were done and revealed an abnormality on the right side of the scrotum. The patient was admitted for a wide inguinoscrotal incision and a right orchidectomy with spermatic cord removal.
